# Acoustic Sensor-Based Soundscape Analysis and Acoustic Assessment of Bird Species Richness in Shennongjia National Park, China

**DOI:** 10.3390/s22114117

**Published:** 2022-05-28

**Authors:** Yanan Hou, Xinwen Yu, Jingyuan Yang, Xuan Ouyang, Dongpu Fan

**Affiliations:** 1 Institute of Forest Resource Information Techniques, Chinese Academy of Forestry, Beijing 100091, China; houyanan@nsu.edu.cn (Y.H.); daylilyau@ifrit.ac.cn (X.O.); fandongpu@ifrit.ac.cn (D.F.); 2 Department of Computer Science Engineering, Chengdu Neusoft University, Chengdu 611844, China; 3 Key Laboratory of Forestry Remote Sensing and Information System, National Forest and Grassland Administration, Beijing 100091, China; 4 Shennongjia National Park Administration, Shennongjia 442421, China; snjbhq@163.com

**Keywords:** acoustic sensors, soundscape analysis, acoustic index, bird species richness, biodiversity monitoring

## Abstract

Passive acoustic sensor-based soundscape analysis has become an increasingly important ecological method for evaluation of ecosystem conditions using acoustic indices. Understanding the soundscape composition and correlations between acoustic indices and species richness of birds, the most important sound source in the ecosystem, are of great importance for measuring biodiversity and the level of anthropogenic disturbance. In this study, based on yearlong sound data obtained from five acoustic sensors deployed in Dalongtan, Shennongjia National Park, we analyzed the soundscape composition by comparing the distributions of the soundscape power in different frequency ranges, and examined the correlations between acoustic indices and bird species richness by means of the Spearman rank correlation coefficient method. The diurnal dynamic characteristics of acoustic indices in different seasons were also described. Results showed that the majority of sounds were in the frequency of 2–8 kHz, in which over 50% sounds were in 2–6 kHz, commonly considered the bioacoustic frequency range. The Acoustics Complexity Index, Bioacoustic Index, and Normalized Difference Soundscape Index were significantly correlated with bird species richness, suggesting that these indices can be used for evaluation of bird species richness; Apparent diurnal dynamic patterns of bird acoustic activities were observed in spring, summer, and autumn; however, the intensity and duration of bird acoustic activities in summer is larger/longer than in spring and autumn.

## 1. Introduction

There is a growing need for cost-effective, scalable ecological monitoring techniques [1] due to global declines in biodiversity [2,3]. The management and conservation of wildlife are increasingly important concerns in a world of limited resources and increasing human population with many wild animal populations under pressure from anthropogenic activities [4]. Conservation areas around the world aim to help conserve animal biodiversity, but it is often difficult to measure conservation success without detailed on-the-ground survey [5]. Repeated on-the-ground surveys can provide the required information to assess animal biodiversity; however, such surveys are limited by being expensive, time-consuming, resource-intensive, invasive [1], can be affected by the biases of individual experts [5], and therefore are not applicable for long-term continuous biodiversity monitoring. An effective and convenient method for evaluating the bird species richness is essential for biodiversity monitoring.

Many animals emit acoustic signals that encode information about their presence and activities. Autonomous sound recording sensors are now available from several companies as small units that are inconspicuous to human [5]. The rapid expansion of this field has been driven by major advances in Passive Acoustic Monitoring (PAM) technology by using acoustic sensors in the wild and has led to growing demand for acoustics expertise in the life sciences [6]. The passive acoustic approach is based on listening techniques elaborated to detect and to monitor individuals without interfering with their behavior [7]. Passive acoustic sensors have become an increasingly important component of the biodiversity survey toolbox [1]. Power consumption, price, and data storage capacity that restricted the deployment longevity of continuous acoustic sensors [8] continue to improve as the technology evolves [9]. The use of PAM as a non-invasive method to sample and monitor ecosystems is increasingly gaining more and more attentions [1,4,6,7,9,10]. PAM was even combined with color perception for representation of urban soundscape quality [11]. With the help of PAM, there is growing interest in soundscape ecology: a recently developed research field that focuses on the study of the temporal and spatial distribution of sound through a landscape, reflecting important ecosystem processes and human activities [12,13,14,15]; soundscape changes can affect animal population and community status [16]. Soundscape ecology uses the acoustic index, a statistic metric summarizing some aspects of the distribution of acoustic energy and information in a recording [17], to rapidly quantify the typical complexity of the biotic songs of a soundscape despite the presence of various noise [18]. In recent years, multiple sound indices have been developed to evaluate the intensity, relative abundance, richness, heterogeneity, and anthropogenic interference on acoustic communities, including the Bioacoustic Index (BI) [19], Acoustic Entropy Index (H) [20], Acoustic Evenness Index (AEI), Acoustic Diversity Index (ADI) [21], Acoustic Complexity Index (ACI) [18], Acoustic Richness Index (AR) [22], Normalized Difference Soundscape Index (NDSI) [14], etc. Acoustic indices have been used for monitoring ecosystem conditions and dynamics [18,23,24,25], and interpretation of environment change [26,27,28,29,30,31]. The acoustic index-based method has become a feasible and promising method to evaluate the status and change in bird population [24,32,33,34].

Birds can provide many ecosystem services [35]. Bird biodiversity can effectively reflect the health status of ecosystems [36], in which bird species diversity is the core content [37]. Bird species richness is one of the most intuitive indicators for evaluating the diversity of the species [37]. Birds make frequent sounds, and their songs are an important part of most wild acoustic communities [38], which are easy to be captured by passive acoustic sensors. Buxton et al. [39] monitored bird migration in Alaska in spring and autumn and found that the ACI index was positively correlated with the number of migratory bird species in this region. In the central Brazilian grassland, ADI was found to have significant positive correlation with bird species richness [36]. Researchers started to use multiple acoustic indices to evaluate bird diversity. Fuller et al. [23] extracted multiple acoustic indices from sound recordings in an eastern Australian forest and found that H, ADI, and NDSI best connected the soundscape with landscape characteristics, ecological conditions, and bird species richness. Mammides et al. [40] found that H, ADI, and AEI obtained from short-term sound data in Yunnan were weakly correlated with bird species richness and diversity.

Shennongjia National Park is rich in primitive forest resources and is the species gene pool and endangered animal and plant shelter [41] in China. Understanding the ecosystem conditions at different levels is of great help to the protection and restoration of the ecosystem. As aforementioned studies suggested, the soundscape method can be a feasible and promising method for monitoring biodiversity and fast evaluation of bird population changes; however, they demonstrated different performances at different places. At present, no studies evaluating the quality of the Shennongjia ecosystem using acoustic data are available. It is very important to find soundscape composition and the correlations between acoustic indices and the bird species richness in Shennongjia National Park to provide a continuous monitoring and fast evaluation method for animal biodiversity. Our study aims to investigate soundscape composition and the diurnal patterns in different seasons of six acoustic indices, and then relate these patterns to bird species richness in five sampling sites of the Shennongjia National Park to further shed light on application feasibility of acoustic indices on rapid assessment of bird species richness.

In this study we deployed five acoustic sensors in Dalongtan, Shennongjia National Park. Based on the yearlong acoustic data obtained from these sensors, we analyzed the soundscape composition by comparing the distributions of the soundscape power in different frequency ranges and examined the correlations between acoustic indices and bird species richness by means of the Spearman rank correlation coefficient method. The diurnal dynamic characteristics of acoustic indices in different seasons were also described.

## 2. Materials and Methods

### 2.1. Overview of the Study Area

**Figure 1:**
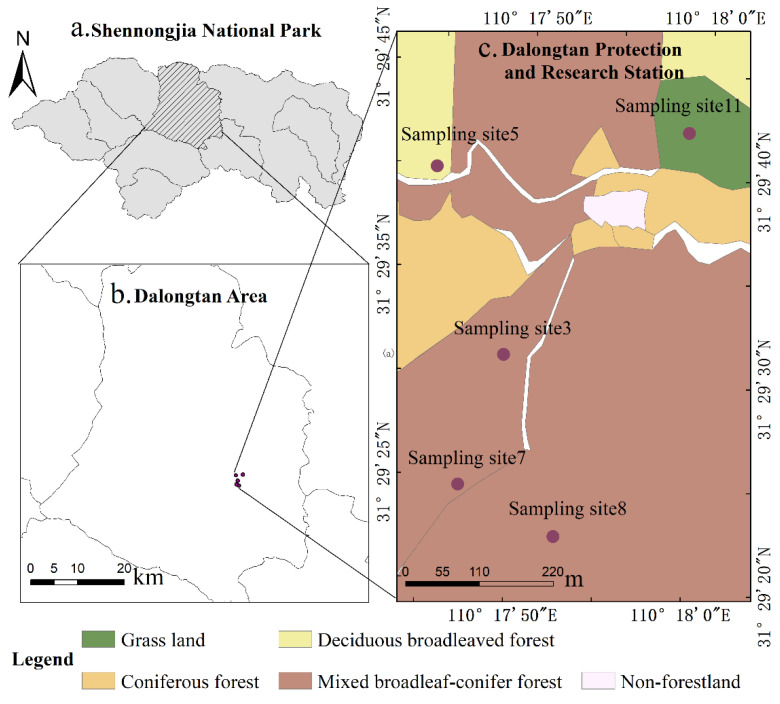
Study area and sampling sites. (**a**) Location of Dalongtan in Shennongjia National Park; (**b**) location of protection and research station in Dalongtan area; (**c**) sampling area and distribution of sampling sites.

Shennongjia National Park, as shown in Figure 1a, is one of the 10 pilot national parks established by the National Development and Reform Commission, China. It is located in 109°56′3.347″–110°36′26.799″ E, 31°17′27.317″–31°36′27.317″ N, in the southwest of the Shennongjia forest area prefecture in Hubei Province, with a total area of about 1325.06 km^2^, where the Shennongjia world natural heritage site, world geopark, national nature reserve, national forest park, 5A level scenic spot, Dajiuhu national wetland park, and Dajiuhu provincial nature reserve are also located [42,43]. There are many high mountains in this area, with an average altitude of 1800 m and the highest peak of Shennongding in central China at an altitude of 3105.4 m. The climate belongs to the transition area between the north subtropical zone to the south and the warm temperate zone to the north. Affected by the mountains, the vertical zonation of temperature was obvious, with an annual precipitation of 800–2500 mm, and the precipitation increases with the increase in altitude.

The studied area was in Dalongtan located almost in the middle of Shennongjia National Park, as shown in Figure 1b covering an area of 192 km^2^. Evergreen coniferous forest (mainly *Abies fargesii*), deciduous coniferous forest (*Larix gmelinii*), deciduous broad-leaved forest (*Betula albosinensis*, *Malus hupehensis*), shrubs, and herbs are mixed in the region with high canopy density and obvious seasonal climate change [41].

The sampling sites, with an area of 2 km^2^, are around the protection and research station for the golden snub-nosed monkey (*Rhinopithecus roxellanae*), a national key protected animal, in Dalongtan area (Figure 1c). There are two bungalows for the working and living of researchers and park rangers, and a wooden walkway leading to the feeding area of monkeys in the station, so there are human activities in this area. There is no anthropized area except the bungalows and walkway. The distribution of different forests are depicted in Figure 1c.

### 2.2. Data Collection

**Figure 2:**
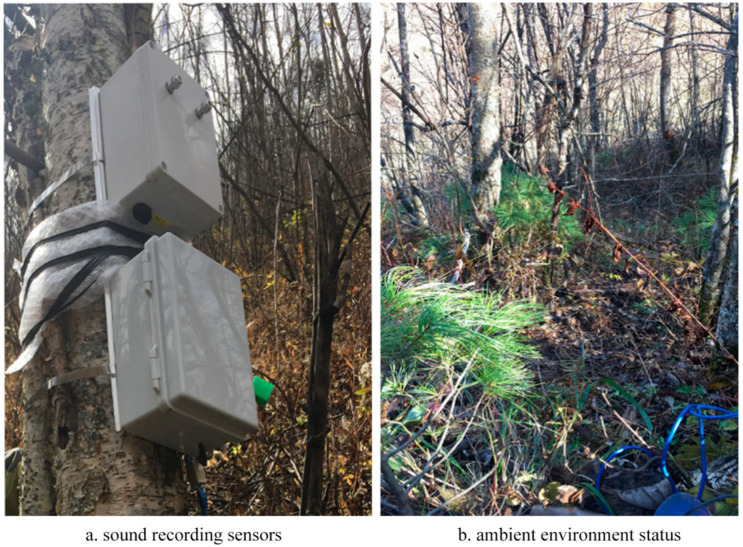
Deployment of sound recording sensors in sampling site and ambient environment status. (**a**) Sound recording sensor was deployed on the trunk 1.5 m above ground; (**b**) ambient environment of sampling site.

A total of 11 acoustic sensors [44] were deployed in the sampling sites in 2018. The acoustic sensor was composed of two microphones, a preamplifier, amplifier, an esp32 control board, and two rechargeable lithium battery packs, as shown in Figure 2a all contained in a waterproof box (upper part) except one battery pack was in the lower box, to prolong working time of the sensor. Detailed parameters of the equipment are shown in Table 1.

The acoustic sensor at each sampling site was configured to record 2 min data every 10 min. A total of 144 data segments were obtained in 24 h These data are stored in mono FLAC audio files with sampling frequency of 64 kHz and sampling depth of 16 bits. These sampling sites covered different habitats in the sample area, such as on top, in the middle, and at the bottom of the slope, beside the stream, valley, and so on. Figure 2b shows the ambient environment of a sampling site. The minimum distance between sampling sites is over 200 m to minimize the pseudo replication of sound (sound of most species in the community will attenuate to none at this distance). Among them, complete 1A data were collected from acoustic sensors in sampling sites 3, 5, 7, and 8, 11, while data from the other sites were incomplete. Therefore, data from sampling sites 3, 5, 7, and 8, 11 as indicated in Figure 1c were selected for analysis in this study.

In order to identify the bird species based on the sound data, we manually listened to the on-site and historical sound recordings with the assistance of the staff of Shennongjia Nature Reserve and some experienced birdwatchers.

### 2.3. Acoustic Data Pre-Processing

Taking solar terms as the 24 time periods of a year, sound data of 3 days before and after each solar term day were selected from Bailu (the fifteenth term, 8 September 2018) to Chushu (the fourteenth term, 23 August 2019). Seasons defined in solar terms were also used in this study. After sorting and screening, a total of nearly 20,000 recording data was obtained.

**Figure 3:**
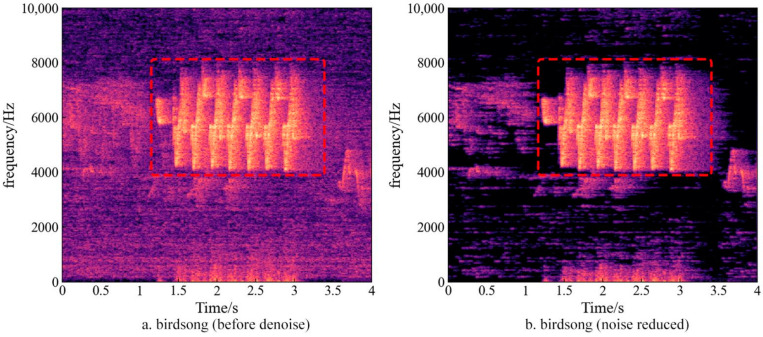
Birdsong spectrum before and after noise reduced. Red rectangle highlighting the frequency spectrum of birdsong. (**a**) Birdsong frequency spectrum before noise reduced; (**b**) birdsong frequency spectrum after noise reduced, noise-reduction processing significantly enhances the noise signal ratio.

Each recording was down-sampled to 44.1 kHz to reduce the amount of data and subsequent computational burden while maintaining the spectral resolution. Spectral subtraction, a commonly used speech enhancement algorithm due to its low computational complexity and strong real-time performance [45], was used to remove the background noise. As shown in Figure 3a, the frequency of birdsong is in the range 4–8 kHz, with concentrated energy distribution (−55~−25 dB). Noise-reduction processing significantly enhanced the signal noise ratio and did not affect the frequency and energy distribution of birdsong (Figure 3b). Low-frequency sound was often considered to be affected by atmospheric noise and can be removed by 1 kHz high-pass filtering. In this study, a 500 Hz high-pass filter was adopted to preserve some low-frequency biological sounds [46].

### 2.4. Soundscape Analysis Method

The term “soundscape” has been used by a variety of disciplines to describe the relationship between a landscape and the composition of its sound [12] since it was first coined in the seventies [47]. ISO defined it as the “acoustic environment as perceived or experienced and/or understood by a person or people, in context” [48], remarking the differences between sonic environment and soundscape [49]. However, in soundscape ecology, soundscape can be defined as the collection of sounds that emanate from a landscape, composed of sounds from physical (geophony), biological (biophony), and anthropogenic (anthrophony or technophony) sources [12,13].

In the overall soundscape, the sound from different sources (birds, insects, animals, natural wind, water flow, rain, human activities such as talking, car honking, etc.) has different frequency range. Each recording was divided in frequency interval of 1 kHz from 1 to 11 kHz, and the power spectral density (PSD) of each frequency interval [50] was calculated using the Matlab pwelch () function [51]. The PSD value for each 1 kHz frequency interval for each recording was then normalized using min-max normalization method and ranged from 0 to 1 for each of the 10 frequency intervals to facilitate comparison across recordings collected at different sites. The normalized PSD was termed soundscape power (SP) [52], it reflects the distribution of signals with different frequencies in the soundscape and was then constructed.

### 2.5. Acoustic Index Calculation

Six commonly used acoustic indices, namely, ACI, ADI, AEI, Bi, H, and NDSI, were selected as the proxies for evaluation of bird species richness. Calculation formula, parameter description and simple introduction of each index was shown in Table 2. AR was not chosen due to the fact that it is greatly affected by the amount of input data.

Indices based on frequency analyses were calculated from a spectrogram computed as the square magnitude of an FFT using window and hop size of 512 and 256 frames.

### 2.6. Correlation Analysis between Acoustic Index and Bird Species Richness

To evaluate the correlation between each acoustic index and bird species richness, the Spearman rank correlation coefficient [53] between the acoustic index and the species richness of birds was calculated for each sampling site.

The Spearman rank correlation coefficient, a commonly used statistic method to assess how well the relationship between two variables, can be described using a monotonic function.

For a sample of size n, the n raw scores $X_{i}$, $Y_{i}$ are converted to ranks $\mathrm{rg}X_{i}$, $\mathrm{rg}Y_{i}$, and $r_{s}$ is computed from:

(8) $$r_{s}=\rho_{\mathrm{rgx},\mathrm{rgy}}=\frac{\mathrm{cov}\left({\mathrm{rg}}_{X},{\mathrm{rg}}_{Y}\right)}{\sigma_{{\mathrm{rg}}_{X}}\sigma_{{\mathrm{rg}}_{Y}}}$$

where ρ denotes the usual Pearson correlation coefficient but applied to the rank variables; $\mathrm{cov}\left({\mathrm{rg}}_{X},{\mathrm{rg}}_{Y}\right)$ is the covariance of the rank variables; $\sigma_{{\mathrm{rg}}_{X}}$ and $\sigma_{{\mathrm{rg}}_{Y}}$ are the standard deviations of the rank variables. Only if all *n* ranks are distinct integers, can it be computed using the popular formula:

(9) $$r_{s}=\rho_{\mathrm{rgx},\mathrm{rgy}}=\frac{\mathrm{cov}\left({\mathrm{rg}}_{X},{\mathrm{rg}}_{Y}\right)}{\sigma_{{\mathrm{rg}}_{X}}\sigma_{{\mathrm{rg}}_{Y}}}$$

(10) $$d_{i}=\mathrm{rg}\left(X_{i}\right)-\mathrm{rg}\left(Y_{i}\right)$$

where *d_i_* is the difference between the two ranks of each observation; the *t*-test was used for the significant test of $r_{s}$.

Eco-acoustics basically assumes that communities with high diversity levels are able to generate a richer acoustic environment [16,19,20]; the acoustic index at the most active moment of acoustic activity in a day was chosen as the representative of that day. Bird species richness, which can be intuitively described by the number of species [52], was estimated by manually listening to the bird sound in the acoustic data, with the help of an experienced birdwatcher.

## 3. Results and Analysis

### 3.1. Frequency Distribution of Sound from Different Sources in the Soundscape

**Figure 4:**
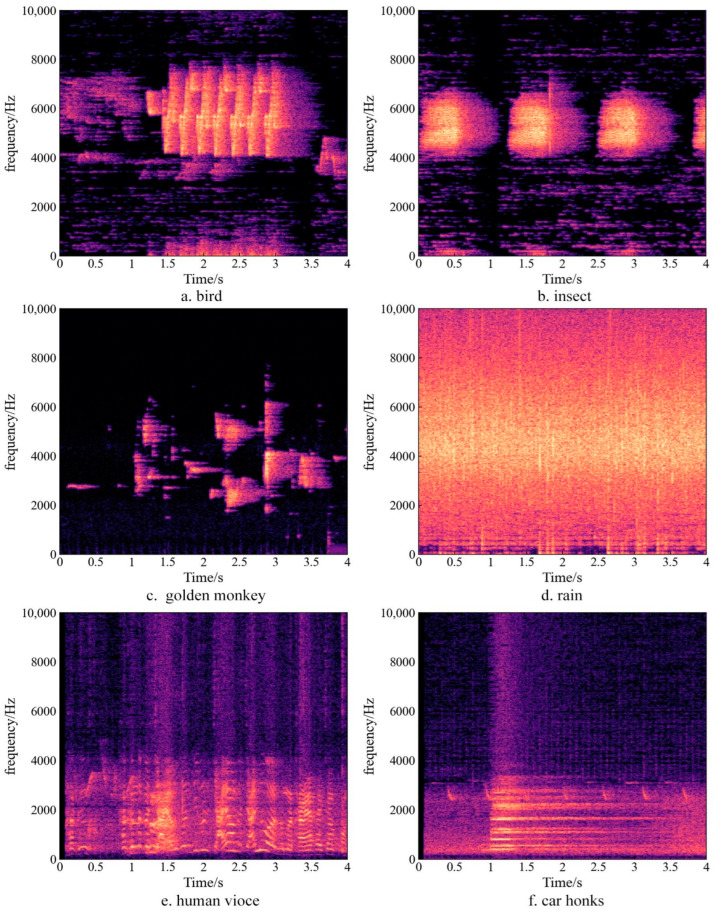
Different frequency characteristics of sounds from different sources: (**a**–**f**).

Frequency characteristics of sounds from different sources were shown in Figure 4. Sounds from bird, insect, and golden monkey demonstrated that the sound energies were concentrating in the frequency distribution. Though the sound frequencies of bird and insect overlapped, the characteristics are quite different in that the time intervals between bird calls were much shorter than that of insect sounds, and insect sounds were usually caught at night while bird calls mostly occurred during daytime. The Sound of the golden monkey was short, frequencies of sounds at the same time jumpily distributed rather than continuously and were much lower than that of bird and insect. Rain sound was the typical geophony, its energy was dispersed in the frequency distribution. Human voices and car honks were lowest in frequency, though they were in a similar lower frequency range, car honk was continuously distributed and not fluctuating in the duration, human voice showed that energy concentrated while talking and obviously weakened and became discrete while breathing.

**Figure 5:**
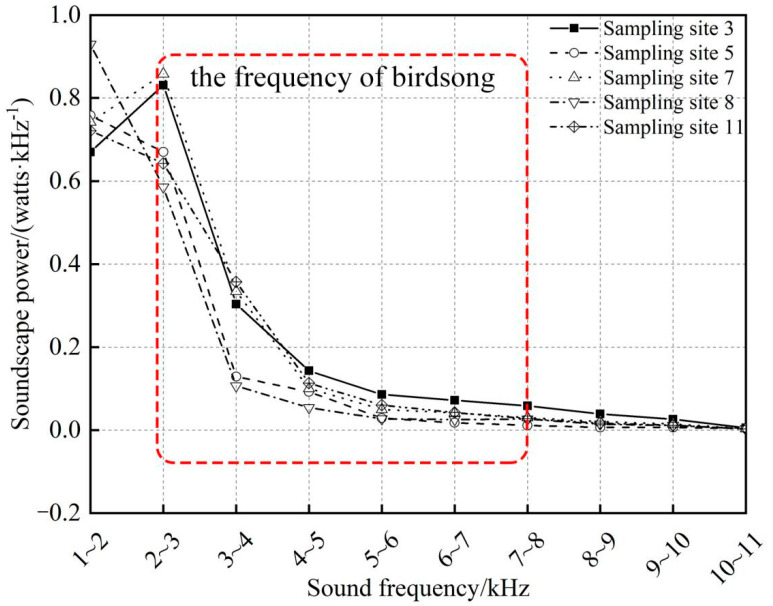
Soundscape power for each of 10 frequency intervals (1–11 kHz) computed from acoustic sensors in five sampling sites. Soundscape compositions of 5 sampling sites are similar, frequency of birdsong is mainly in range 2–8 kHz, as indicated by the red rectangle.

PSD for each 1 kHz frequency interval of each recording at each sampling site was calculated, and normalized PSDs produced SP values ranging from 0 to 1. The whole year SP was derived from the mean value of all sound data in each frequency range, as shown in Figure 5. To examine the influence of sound within each frequency interval to the overall soundscape, the ratio of SP in each frequency interval to the overall SP was calculated. The results were shown in Table 3.

As shown in Figure 5, the majority of sounds in the soundscape were in 2–8 kHz (Table 3), which was in accordance with birdsong frequency. In which, over 30% of sounds were in the range 2–3 kHz, and 20% were in the range 3–6 kHz. High SP values at sampling sites 3 and 7 were in frequency the range 2–3 kHz, others were in the range 1–2 kHz. Sounds in high-frequency (8–11 kHz) had a very low proportion and were not an important part of the soundscape.

Soundscape categorizes sounds produced in ecosystems as biological (biophony), anthropogenic (anthrophony), and natural sounds (geophony) [13], in which the anthrophony, such as human talking and car honks are most prevalent between 1 and 2 kHz [13], just as shown in Figure 4e,f. Results in Figure 5 and Table 3 showed that sounds in range of 1–2 kHz were in considerable proportion, suggesting that there was remarkable anthropogenic interference [52]. Manually listening to the sounds also verified that there were sounds from human activities because all sampling sites were very close to the residential area of the protect and research station.

### 3.2. Correlation between Acoustic Index and Bird Species Richness

To analyze the correlation between acoustic indices and bird species richness, six indices from each recording segment were calculated. Unlike the calculation of other indices using noise-reduced sound, data without noise-reduction processing were adopted in the calculation of NDSI to avoid the change in frequency distribution caused by noise reduction since NDSI identifies sound category by frequency range. Results of the correlation between acoustic indices and bird species richness at different sampling sites were shown in Table 4. The Bi index showed an extremely significant correlation with bird species richness at sampling sites 5 and 7 and the correlation coefficient reached 0.610 at sampling site 5. The ACI showed a highly significant correlation with bird species richness at sampling sites 3, 5, and 11, with the highest correlation coefficient of 0.736 at sampling site 11. The AEI index and h-index, showed a highly significant correlation with bird species richness at sampling site 11, with correlation coefficients of −0.474, 0.475, respectively. NDSI indices were highly significantly correlated with bird species richness at sampling sites 5, 7, and 8, with the highest correlation coefficient of 0.669; at sampling site 11, many high NDSI values appeared, which was due to insect vocalizations rather than birds, and thus the correlations were weak. No significant correlation was found between the ADI index and species richness of birds.

To minimize the influence of sounds from nature, human activity, and other non-avian biological sounds on the evaluating effect of bird species richness, the above-mentioned sounds were removed manually after carefully listening to the sound data. However, NDSI is essentially an index to estimate the level of anthropogenic disturbance on the soundscape by computing the ratio of human-generated to biological acoustic components found in field collected sound data, only the natural sounds were removed for computing of the NDSI index. After processing, six acoustic indices were computed again and optimized acoustic indices obtained. The Spearman rank correlation coefficients between the optimized acoustic indices and the bird species richness are shown in Table 5. Correlations of almost all indices with bird species richness increased compared with the results in Table 4. The ACI index showed a significant correlation with bird species richness at sampling site 8 and an extreme significant correlation in other sites, with the highest coefficient of 0.824 at sampling site 11. Bi showed a highly significant correlation with bird species richness at sampling sites 5, 7, and 11, while correlation between NDSI and species richness was extremely significant at sampling sites 5 and 7, significant at sampling sites 3 and 8, and not significant and remained the same as in Table 4 at site 11, indicating that it was less affected by geophony. The AEI retained the same extremely significant correlation with bird species richness at sampling site 11 but changed to significant correlation from not significant at sample 3. No significant correlation was observed for the other sound indices.

Comprehensive analysis of the results in Table 4 and Table 5 showed that Bi, ACI, and NDSI indices correlated well with bird species richness, and can be suitable candidate indices for evaluation of bird species richness. Other acoustic indices demonstrated poor correlation with bird species richness and are not applicable for assessment of bird species richness.

### 3.3. Diurnal Dynamic Characteristics of Acoustic Indices in Different Seasons

**Figure 6:**
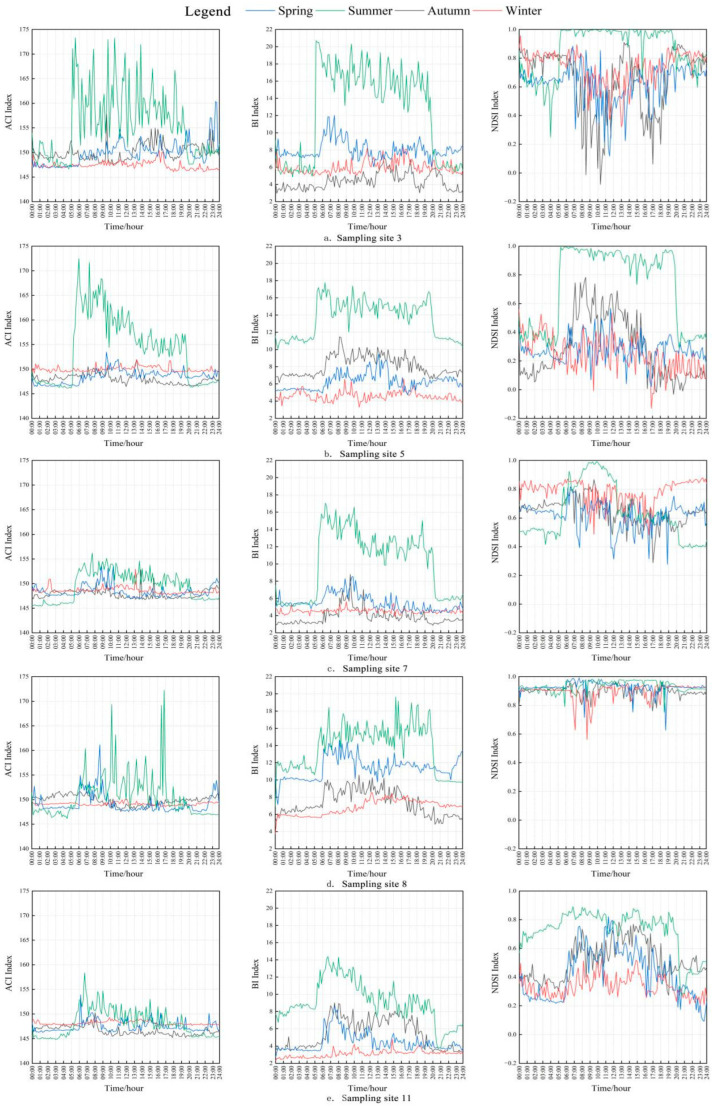
Diurnal dynamic characteristics of acoustic indices in different seasons at different sampling sites (**a**–**e**). The subfigures from column 1 to 3 are dynamics of ACI, Bi and NDSI in 5 sampling sites. Bird calls were active in summer, there were obvious diurnal patterns of acoustic indices dynamics in sampling sites.

To analyze the difference of acoustic activities of birds in different seasons, the performances of significantly correlated Bi, ACI, and NDSI indices in different seasons at five sampling sites were examined as shown in Figure 4, in which each acoustic index value is the average of acoustic index values at the same moment on all days in that season (Figure 6).

Figure 6 shows that the performances of Bi and ACI at each sampling site were consistent across seasons. All index values increased rapidly at dawn, remained high during the day, and declined in the evening and kept stable all night, demonstrating an obvious diurnal dynamic pattern, which was in accordance with the habits of bird dawn chorus around 05:00 and dusk chorus around 19:00 [54]. In summer, Bi and ACI had higher values than in other seasons, indicating that birds are more active than in other seasons. In spring and autumn, the two indices also showed similar patterns, but the variation dynamic of each index was weaker than that in summer. In winter, Bi and ACI values were at low levels throughout the day and did not change significantly.

The diurnal performance of NDSI was relatively less consistent in different seasons compared with Bi and ACI, showing apparent fluctuations. In summer, the most significant variation times of NDSI were 05:00 and 19:00 in most sampling sites, similar to that of Bi and ACI. At sampling site 11, a high value continued from 0:00 to 05:00, which can be deemed as influence of nearby insect activities during summer at night which was verified by careful listening to the sound data. In other seasons, only sampling site 11 had a similar diurnal variation pattern to Bi and ACI, and there was no obvious diurnal variation pattern found in other sampling sites. As we can observe in Figure 4, NDSI values in four seasons were high at sampling sites 3, 7, and 8, and the value at sampling site 8 was close to the highest value, suggesting a high biophony in this site. Our field survey showed that these three sampling sites were close to water streams, and the spectrum of sound data indicated that the frequency of the stream sound was in range of 3–6 kHz, coincident with frequency range of biophony, which was the reason of the high NDSI value. The NDSI value also fluctuated downward at sampling site 3, this was because human (mainly researchers and park rangers) activities frequently occurred in site 3, which was close to the research base for the golden snub-nosed monkey, while bird activity was relatively less active in spring, autumn, and winter.

Seasonal differences in these acoustic indices indicated that bird call activity frequently occurred in summer. Typically, birds began their dawn ensembles and chorus around 05:00 in the morning and 19:00 in the dark in summer. There were some bird activities in spring but they were less active than in summer. Bird dawn chorus in spring and autumn was delayed while the dark chorus ended earlier than in summer. Bird activity was rarely captured in winter. This seasonal difference is also in accordance with the seasonal change pattern of the bird population. Birds breed mostly in spring, and the population increased due to the bearing of new individuals, then declined as some individuals died during their growth, and always became the lowest at the beginning of the next breeding season [55]. Meanwhile, according to the historical field trip record in Shennongjia [41,42], a vast majority of birds in Shennongjia are resident birds, followed by summer migratory birds, winter migratory birds, and passing birds. The species richness of birds was observed in summer (148 species), autumn (77 species), spring (72 species), and winter (28 species) in descending order. This seasonal species richness difference verified the seasonal difference of acoustic indices in some extents. The temporal dynamics of acoustic indices through the long term in this research revealed that, compared with that of the short term [23,40], the acoustic indices obtained over the long term were able to more accurately capture the diurnal dynamic characteristics of acoustic indices and were more suitable for bird species richness assessment.

## 4. Discussion

In this study, the overall soundscape power value was analyzed to obtain the composition of the soundscape in the sampling area. The background noise of the recording device with frequency from 1 to 2 kHz was not the real composition of the soundscape, but its influence on the soundscape could not be ignored. Almost half of the sound in the soundscape was in the range of 2 to 4 kHz, and this frequency range was dominated by bird calls. Birds have dawn and dusk chorus habits; thus it was expected that due to dawn and dusk chorus (around 5:00 and 19:00), acoustic activities should obviously increase. However, in this study, compared with other durations of the day (such as noon), the acoustic index around 19:00 did not increase significantly, on the contrary it demonstrated a sharp decline. Further attention needs to be focused on bird chorus activity and the performance of acoustic index at dusk to find out the reasons for the increase acoustic index at the dawn chorus no increase at dusk, and decrease after dark chorus for the acoustic indices. Sounds in range of 4 to 5 kHz occurred mainly at night. High-frequency sound (6–8 kHz), mainly from insect activities, only accounted for a small proportion of the overall soundscape, indicating that insect species richness and abundance were small in the sampling area. In this study, a whole year of recording data were adopted for the analysis of soundscape and acoustic indices. In the following research, longer time series recording data will be used to examine more detailed and accurate soundscape composition, and spatio-temporal dynamics of acoustic indices since the recording devices are still working and deployed to a larger area of Shennongjia National Park; this will further better understanding of bird call activity, migration, and reproduction, and the bird bio-diversity dynamics in the ecosystem.

The eco-acoustic method is an efficient approach to evaluate bird species richness. This research showed that ACI and Bi significantly correlated with the bird species richness in Shennongjia National Park, and this result was consistent with the conclusions of other studies [18,19]. Comparative analysis of diurnal variation in acoustic indices across seasons showed that the bird dawn ensembles and chorus started earlier while dusk chorus ended later in summer than in spring and autumn, indicating that bird call and chorus are closely related to breeding, predation period, and geographic activity [54]. Moreover, temporal dynamics of bird calls and the interval of bird activity in the sampling area can be extracted from the variation patterns of acoustic indices, suggesting that the soundscape method based on the acoustic index can effectively reflect the change in bird species richness. This is of great help to the scheme making for biodiversity monitoring.

During the extraction of bird acoustic indices, it is very important that natural sounds (geophony), human activity sounds (anthrophony), and other non-avian biological sounds must be excluded since they produce obvious interference to the computing of acoustic indices. Among them, geophony, especially weather conditions, had a greater influence on the bird acoustic index [56]. Heavy rain and strong wind can produce sounds of similar frequency range with biological sounds, e.g., the correlation coefficients between ACI indices and bird species richness in sampling sites 3 and 5 clearly increased when the interference sounds from weather and water were removed. Many studies also recorded weather conditions while collecting sounds [21,57] to enable the automatic exclusion of weather disturbance. For the selection of eco-acoustic indices, a cluster analysis based on PCA has proved to be a robust method in determining groups of recordings with a similar sound environment by highlighting the periods of the day corresponding to higher eco-acoustic dissimilarity [58]. This method can be used in our future work to provide a more persuasive result.

One researcher suggested that NDSI can be used to describe the ratio of biophony [52]. In this study, NDSI correlated significantly with bird species richness, and at sampling sites 5 and 11, both biological and abiotic sounds could be distinguished with NDSI; while at sampling sites 3, 7, and 8, there was the influence of water sounds with frequency in the range 36 kHz, which is coincident with the range of biophony, resulting in high NDSI values indicating that NDSI may identify geophony as biophony; therefore it may not be a suitable assessment proxy in this case. However, compared with the difficulty of manual listening to the sound data, using NDSI to identify anthrophony and then remove it is much easier and efficient, improving the evaluation effects of the other acoustic indices. This requires detailed planning in advance for sound data acquisition and suitable sampling site selection to avoid interference sound such as sound from water flow.

## 5. Conclusions

In this study, soundscape composition and the frequency distribution of sounds from different sources in Dalongtan of Shennongjia National Park were analyzed by calculating the soundscape power value using long-term sound data. The majority of sounds in the soundscape are in the range of 2 to 8 kHz; 1–2 kHz mainly comes from background noise of the recording sensors, over 50% sounds are in 2–6 kHz, which is usually considered the frequency of biological sounds mainly from birds; only a small portion of sounds were from mammals such as golden monkey and insects.

Six acoustic indices, BI, ACI, NDSI, ADI, AEI, and H, were extracted from yearlong sound data obtained by our acoustic sensor equipment in Dalongtan, Shennongjia National Park. ACI, BI, and NDSI can better reflect the richness of local bird species, and the temporal dynamics of the acoustic indices can effectively reflect the changes in bird activities in the area in different time periods, proving that using sound data from acoustic sensors to assess bird diversity is promising in its effectiveness and efficiency, and it is non-invasive, human-independent, and economically efficient.

ACI and Bi had obvious diurnal dynamic patterns of increasing rapidly at dawn, remaining high during the day, declining in the evening, and being stable all night across four seasons. In summer, Bi and ACI had higher values than in other seasons and fluctuated drastically. In spring and autumn, the two indices were low and showed similar patterns, but the variation dynamic was weaker. In winter, Bi and ACI values were at low levels throughout the day and did not change significantly. There were also seasonal patterns of bird singing activity. In summer, bird call activity frequently occurred, and birds began dawn chorus around 05:00 in the morning and dark chorus around 19:00. Bird call activity obviously declined in spring and autumn, dawn chorus delayed, and dark chorus ended earlier than in summer. Rare calling activity occurred in winter. Influenced by the running water sound, NDSI did not show a similar diurnal and seasonal pattern. The diurnal and seasonal patterns of acoustic indices would be of great importance to the scheme making for biodiversity monitoring.

Performances on the assessment of bird species richness of almost all acoustic indices were increased after removing sounds from human activities, natural environment sounds, and non-avian animals; NDSI may identify geophony as biophony, and therefore may not be a suitable assessment proxy in this case. Therefore, when using acoustic indices for evaluation of bird biodiversity, influence of geophony, anthrophony, and non-avian sound should be carefully considered, and it would be of great help to remove these influences in advance.

The recording data that conserved the acoustic originality of the ecosystem can be easily saved, repeatedly analyzed, and ready to be processed and analyzed using newly developed acoustic methods. This research was conducted in a very small area and only dealt with the time series data for soundscape and acoustic indices analysis, though the results were enlightening, it may not be a good representation for Shennongjia National Park. Further study in a wider area and investigation on the spatio-tempo relevance and patterns of soundscape and acoustic indices are required, and these works are currently being undertaken. Information obtained from these studies will be very helpful not only for fast assessment of biodiversity, but also to help park authorities to make a biodiversity monitoring and conservation plan.

## Figures and Tables

**Table 1:** Technical parameters of the acoustic sensors.

Items	Parameter
Channel	Mono, stereo
Format	Wav, flac
Sonic range	20 Hz–48 kHz
Sampling rates (Hz)	8 k–96 k
Storage	2 card slots, each up to 128 GB (SDHC) or 512 GB (SDXC); Maximum capacity up to 1.0 TB (two 512 GB SDXC cards);
Shell	Waterproof

**Table 2:** Acoustic indices calculation method.

Name	Calculation Method		Symbolic Interpretation	Usage
BI [19]	$\overset{N}{\underset{n=1}{\sum }}\left(S_{mean}-S_{min}\right)\times f$	(1)	$S_{mean}$: the mean spectrum across f; $S_{min}$: the minimum dB value of this mean spectrum; f: list of the frequencies of the spectrogram.	Used to compare frequency amplitude spectra to detect or infer differences in avian community composition among sites [19].
ACI [18]	$\overset{q}{\underset{l=1}{\sum }}\overset{m}{\underset{j=1}{\sum }}\frac{\sum_{k=1}^{n}\left|I_{k}-I_{k+1}\right|}{\sum_{k=1}^{n}I_{k}}$	(2)	q: number of spectrums; m: number of time steps in bin; n: the number of subsets in a single frequency bin; $I_{k}$: the intensity in a single frequency bin.	Used to determine changes in behavior and composition of a vocalizing community and consequently to better monitor animal dynamics in a quick way [18].
ADI [21]	$\overset{S}{\underset{i=1}{\sum }}p_{i}\ln p_{i}$	(3)	$p_{i}$: the proportion of the signals in each bin above a threshold (−50 dBFS), $p_{i}\leq p_{i+1}$; S: the number of spectrograms.	Representing sound diversity used to compare the soundscape differences between different times among different sites [21].
AEI [21]	$\frac{2\sum_{i=1}^{S}ip_{i}}{S\sum_{i=1}^{S}p_{i}}-\frac{S+1}{S}$	(4)		Used to compare the frequency differences between sounds from different land usages [21].
H [20]	$H_{t}\times H_{f}$ $H_{f}=-\overset{N}{\underset{f=1}{\sum }}S\left(f\right)\times {log}_{2}\left(S\left(f\right)\right)\times {log}_{2}{\left(n\right)}^{-1}$ $H_{t}=-\overset{n}{\underset{t=1}{\sum }}A\left(t\right)\times {log}_{2}\left(A\left(t\right)\right)\times {log}_{2}{\left(n\right)}^{-1}$	(5) (6)	n: length of the signal in number of digitized points; A(t): probability mass function of the amplitude envelope; S(f): probability mass function of the mean spectrum calculated.	Derived from the Shannon information statistic, higher values of H indicates richer habitats [20].
NDSI [14]	$\frac{B_{sound}-A_{sound}}{B_{sound}+A_{sound}};\;-1\leq NDSI\leq 1$	(7)	$B_{sound}$: biophony; $A_{sound}$: anthrophony; NDSI < 1 the levels of anthrophonies is higher; NDSI < 1 the levels of biophonies is higher.	Used to estimate the level of anthropogenic disturbance on the soundscape [14].

**Table 3:** Percentage of total soundscape power for each of 10 frequency intervals (1–11 kHz) computed from sound recordings at five sampling sites, the unit of frequency is kHz.

Sampling Sites	Proportion of SP in Soundscape (%)									
	1~2	2~3	3~4	4~5	5~6	6~7	7~8	8~9	9~10	10~11
Site 3	30.0	37.2	13.6	6.4	3.8	3.8	2.6	1.8	1.8	0.3
Site 5	43.9	38.9	7.5	5.4	1.7	1.0	0.7	0.4	0.4	0.2
Site 7	33.8	39.1	15.2	4.6	2.3	1.9	1.4	0.9	0.7	0.2
Site 8	52.1	32.8	6.0	3.0	1.5	1.4	1.5	0.8	0.6	0.2
Site 11	36.1	32.2	17.9	5.7	3.0	2.1	1.4	0.8	0.6	0.1

**Table 4:** **The** Spearman rank correlation coefficient between acoustic indices and avian species richness at each sampling site.

Sampling Sites	BI	ACI	ADI	AEI	H	NDSI
Site 3	0.226	0.544 **	0.118	−0.010	−0.383	0.485 *
Site 5	0.610 **	0.698 **	0.127	−0.154	−0.192	0.698 **
Site 7	0.595 **	0.281	−0.002	0.010	−0.270	0.669 **
Site 8	0.439 *	0.381	−0.232	0.260	−0.447 *	0.503 **
Site 11	0.351 *	0.736 **	0.414 *	−0.474 **	0.475 **	0.225

** represents a significant correlation at the 0.01 (two-sided) level, * represents a significant correlation at the 0.05 (two-sided) level.

**Table 5:** **The** Spearman rank correlation coefficients between acoustic indices and bird species richness at different sites after geophony elements removed.

Sampling Sites	BI	ACI	ADI	AEI	H	NDSI
Site 3	0.321	0.824 **	−0.312	0.466 *	−0.372	0.509 *
Site 5	0.617 **	0.706 **	−0.039	0.116	−0.311	0.696 **
Site 7	0.617 **	0.751 **	−0.049	0.194	−0.404	0.645 **
Site 8	0.322	0.581 *	−0.131	0.121	−0.427 *	0.481 *
Site 11	0.661 **	0.806 **	0.355 *	−0.450 **	0.187	0.225

** represents a significant correlation at the 0.01 (two-sided) level, * represents a significant correlation at the 0.05 (two-sided) level.

## Data Availability

Not applicable.
